# Heterochromatin variation and LINE-1 distribution in *Artibeus* (Chiroptera, Phyllostomidae) from Central Amazon, Brazil

**DOI:** 10.3897/CompCytogen.v11i4.14562

**Published:** 2017-09-14

**Authors:** Érica Martinha Silva de Souza, Maria Claudia Gross, Carlos Eduardo Faresin e Silva, Cibele Gomes Sotero-Caio, Eliana Feldberg

**Affiliations:** 1 Programa de Pós-graduação em Genética, Conservação e Biologia Evolutiva, Instituto Nacional de Pesquisas da Amazônia, Av. André Araújo, 2936, Aleixo, 69.060-001 Manaus, AM, Brazil; 2 Laboratório de Genética Animal, Instituto Nacional de Pesquisas da Amazônia, Av. André Araújo, 2936, Aleixo, 69.060-001 Manaus, AM, Brazil; 3 Universidade Federal da Integração Latino Americana, Laboratório de Genética, Av. Tarquínio Joslin dos Santos, 1000, Jardim Universitário, 85857-190, Foz do Iguaçu, PR, Brazil; 4 Department of Biological Sciences, Texas Tech University, Lubbock, TX, USA 79409; 5 Laboratório de Genética e Citogenética Animal e Humana, Departamento de Genética, Universidade Federal de Pernambuco, Av. da Engenharia s/n; Cidade Universitária; CEP:50740-600; Recife-PE, Brazil

**Keywords:** Bats, chromosomes, cytogenetics, FISH, repetitive DNA, Stenodermatinae

## Abstract

Species in the subgenus Artibeus Leach, 1821 are widely distributed in Brazil. Conserved karyotypes characterize the group with identical diploid number and chromosome morphology. Recent studies suggested that the heterochromatin distribution and accumulation patterns can vary among species. In order to assess whether variation can also occur within species, we have analyzed the chromosomal distribution of constitutive heterochromatin in *A. planirostris* (Spix, 1823) and *A. lituratus* (Olfers, 1818) from Central Amazon (North Brazil) and contrasted our findings with those reported for other localities in Brazil. In addition, Ag-NOR staining and FISH with 18S rDNA, telomeric, and LINE-1 probes were performed to assess the potential role that these different repetitive markers had in shaping the current architecture of heterochromatic regions. Both species presented interindividual variation of constitutive heterochromatin. In addition, in *A. planirostris* the centromeres of most chromosomes are enriched with LINE-1, colocated with pericentromeric heterochromatin blocks. Overall, our data indicate that amplification and differential distribution of the investigated repetitive DNAs might have played a significant role in shaping the chromosome architecture of the subgenus Artibeus.

## Introduction

Currently, three species of large body size Artibeus (subgen. Artibeus Leach, 1821) are found in the Brazilian Amazon region: *A. obscurus* (Schinz, 1821), *A. lituratus* (Olfers, 1818), and *A. planirostris* (Spix, 1823) (Marques-Aguiar 2007, Gardner 2008). These bat species occur in sympatry in most Brazilian environments, and display considerable morphological variation along their geographic distribution. The overlapping measurements of external morphological characters can still lead to misidentification between *A. planirostris* and *A. obscurus* in the field. On the other hand, *A. lituratus* and *A. planirostris* are easily distinguishable morphologically (Gardner 2008). Nevertheless, cranial features and a more detailed examination of voucher specimens usually provide diagnostic characters for species identification (Ortega and Castro-Arellano 2001, Haynes and Lee 2004, Lim et al. 2004).

Cytogenetic studies in all species of the subgenus Artibeus revealed a conserved karyotype, with diploid number (2n) of 30 chromosomes for females and 31 for males, with fundamental number, FNa = 56 (Baker 1967, Souza and Araújo 1990, Noronha et al. 2001, Santos et al. 2002, Baker et al. 2003, Calixto et al. 2014). The 2n difference between females and males is due to a XX/XY_1_Y_2_ multiple sex chromosome system shared by most species of the subfamily Stenodermatinae P. Gervais, 1856 (Tucker and Bickham 1986, Noronha et al. 2001, Rodrigues et al. 2003, Pieczarka et al. 2013). Although the overall patterns of classical cytogenetic markers (including G- and C- banding, and Ag-NOR staining) are fairly well investigated, variation of constitutive heterochromatin (CH) distribution was just recently reported among *Artibeus* species (Lemos-Pinto et al. 2012). In their work, Lemos-Pinto et al. (2012) investigated the CH distribution in the karyotypes of *Artibeus* from the state of Pernambuco (Northeast Brazil), and proposed that the CH patterns were species-specific. However, although independent studies focused on species cytogenetic characterizations at the local level, no study has targeted the detection of interindividual CH variation in *Artibeus* within and among different Brazilian regions.

Chromosomal evolution, including variation in the patterns of CH distribution is usually associated with distinct repetitive DNA dynamics. Therefore, *in situ* mapping of repetitive markers (e.g., 18S rDNA and telomeric sequences and interspersed repetitive elements) can significantly contribute to the understanding of the evolution of genome architecture, as well as to the identification of intraspecific polymorphism in karyotypes otherwise conserved (Baker and Bickham 1980, Morielle and Garcia 1988, Varella-Garcia et al. 1989, Souza and Araújo 1990, Baker et al. 2003, Lemos-Pinto et al. 2012). Howerver, there is still a lack of studies correlating the localization of CH and repetitive elements in bats. This is particularly true for transposable elements (TEs), despite their significant incidence in vertebrate genomes, and their potential to drive heterochromatin formation (Gentles et al. 2007, Chalopin et al. 2015, Sotero-Caio et al. 2017). For example, although LINE (Long Interspersed Nuclear Element) retrotransposons are the most prevalent TEs in mammals, their chromosomal distribution were described for only four bat species (Parish et al. 2002, Sotero-Caio et al. 2015).

In the present study, we investigate whether there is CH variation within Central Amazon populations (North Brazil) of two *Artibeus* species (*A. planirostris* and *A. lituratus*), as well as CH variation among representatives from Amazonian and other Brazilian regions. Furthermore, we have mapped rDNA and telomeric sequences on the karyotypes of both species to assess whether these sequences contribute to the architecture of centromeres and other positive heterochromatin blocks. As our final goal, we investigated the chromosomal distribution of LINE-1 sequences in *A. planirostris* chromosomes to i) compare with patterns described for other phyllostomid species, and ii) correlate the distribution of these sequences with the CH pattern observed for individuals in the same population.

## Materials and methods

The specimens used in this investigation were collected during expeditions conducted in 2009. The sampling locations were not within protected areas, and *Artibeus* species used in this study are not listed as endangered at national or local levels. Our sampling included specimens of *A. planirostris* collected in an urban fragment at the National Institute of Amazonian Research (INPA) (03°05'51.1"S, 59°59'8.4"W), and at “Bons Amigos” Farm (Km 14 of BR 174; 02°50'37"S, 60°03'58"W). Furthermore, we collected individuals of *A. lituratus* at “Bons Amigos” Farm, Amazonas State, Brazil (Table 1). Voucher specimens and cytological material were deposited at the “Laboratório de Genética Animal” at INPA.

**Table 1.** List of specimens and respective methodologies applied in the present study. Sampling localities for each voucher are given in the last column.

Species	Voucher ID	Sex	Giemsa Staining	C-banding	Ag-NOR Staining	18S FISH	Telomeric FISH	LINE-1 FISH	Sampling Site
*A. planirostris*	EMS05	♂	X	X	–	–	–	–	Urban fragment at INPA
	EMS06	♂	X	X	X	–	–	–	Urban fragment at INPA
	EMS07	♂	X	X	X	X	X	–	Urban fragment at INPA
	EMS09	♀	X	X	X	X	X	X	Urban fragment at INPA
	EMS10	♂	X	X	–	–	–	X	Urban fragment at INPA
	EMS18	♀	X	X	X	X	X	–	“Bons Amigos” Farm
	EMS14	♂	X	X	X	X	X	–	“Bons Amigos” Farm
	EMS17	♂	X	X	X	–	–	–	“Bons Amigos” Farm
*A. lituratus*	EMS15	♀	X	X	X	X	X	–	“Bons Amigos” Farm
	EMS16	♂	X	X	X	X	X	–	“Bons Amigos” Farm
	EMS19	♂	X	X	X	X	–	–	“Bons Amigos” Farm

Mitotic chromosomes were obtained from bone marrow cells using the *in vivo* method (Lee and Elder 1980, Varella-Garcia and Taddei 1989). C-banding patterns and nucleolus organizing region (NOR) locations were determined according to Sumner (1972), and Howell and Black (1980), respectively. The FISH probes were prepared by PCR using primers to amplify the 18S ribosomal gene (18SF, 5’ CCGCTTTGGTGCTCTTGAT 3’; 18SR, 5’ CCGAGGACCTCATAAACCA 3’) (Gross et al. 2010), the telomeric sequences (TTAGGG)_n_ (Ijdo et al. 1991), and LINE-1 (L1R, 5’ ATTCTRTTCCATTGGTCTA 3’; L1F, 5’ CCATGCTCATSGATTGG 3’) (Waters et al. 2004) (Table 1). The PCR products were labeled by nick translation using biotin kit (Bio-Nick ROCHE). FISH procedures followed Pinkel et al. (1986) with modifications: mitotic chromosomes were denatured in 70 % formamide/0.6X SSC (pH 7.0) for 5 minutes at 70 °C; the hybridization mix applied per slide contained 200 ng of probe, 10 % dextran sulfate, 2 X SSC and 50 % formamide in a final volume of 40 µl. Slides were incubated overnight at 37 °C. Post-hybridization washes were carried out at 42 °C in 15% formamide/0.2X SSC for five minutes. Detection was performed with avidin-FITC (fluorescein isothiocyanate) conjugate (Sigma, St Louis, MO, USA), followed by counterstaining with Propidium Iodide (0.2%) and mounting in Vectashield (Vector, Burlingame, CA, USA).

The chromosomes were analyzed using an Olympus BX51 microscope, and the metaphases were captured with an Olympus DP70 digital camera using IMAGE-PRO MC 6.0 software. The images were processed using ADOBE PHOTOSHOP CS3 program, and the chromosomes were measured using the IMAGE J (Schneider et al. 2012). The chromosomes were classified as metacentric (m), submetacentric (sm), subtelocentric (st) and acrocentric (a), in descending size order (Levan et al. 1964). The fundamental number was based on the number of autosomal arms (FNa), as described by Gardner and Patton (1976).

C-banding reports from Souza and Araújo (1990), Rodrigues et al. (2003), and Lemos-Pinto et al. (2012) were assessed to detect inter- and intraspecific CH variation among specimens from different Brazil regions.

## Results and discussion

### Classical Cytogenetics and Constitutive Heterochromatin Variation

**Figure 1.**
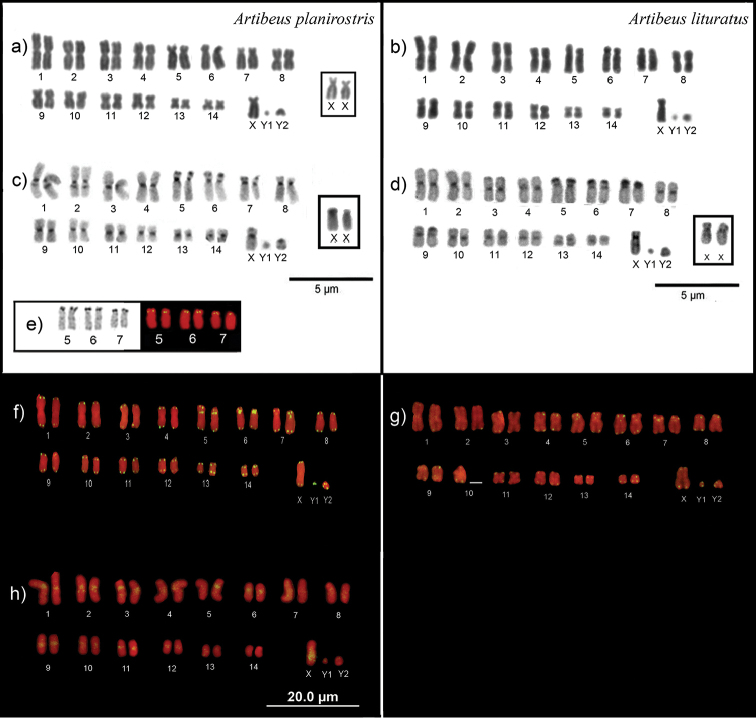
Karyotypes of *A. planirostris* (**a, c, e, f, h**) and *A. lituratus* (**b, d, g**). Conventional staining (**a, b**); C-banding patterns (**c, d**); Ag-NOR staining (left) and FISH with rDNA 18S (rigth; **e**), FISH using telomeric repeats as probes (**f, g**), FISH with probes from the open reading frame (ORF) II of LINE-1 from *A. planirostris* (**h**).

Classical Giemsa staining did not uncover structural variation between the karyotypes of *A. planirostris* and *A. lituratus* from Amazonas. Both species have the same diploid (2n = 30/31, XY_1_Y_2_) and fundamental numbers (FNa = 56), with 11 metacentric and three subtelocentric chromosome pairs (22m+6st+XX/XY_1_Y_2_). The X chromosome was a medium submetacentric, Y_1_ had a dot-like morphology, and Y_2_ was a small acrocentric (Fig. 1a, b).

Despite having the same karyotype, slight differences of constitutive heterochromatin distribution were observed, especially for sex chromosomes, between *A. planirostris* and *A. lituratus*. C-banding revealed CH in the centromeric region of all autosomes of both species. Additionally, in *A. planirostris* small heterochromatic blocks were observed in the proximal region of long arms on two metacentric chromosomes (1^st^ and 2^nd^ pairs), as well as in the distal region of short arms on three subtelocentric pairs (5^th^, 6 ^th^ and 7 ^th^), which are adjacent to the location of active Ag-NORs. The Y_1_ chromosome was euchromatic, and the Y_2_ had centromeric heterochromatin and additional blocks on the long arms. Likewise, the X chromosome showed centromeric heterochromatin and blocks on the short arms. The long arm of the X chromosome however, was not particularly enriched with heterochromatin (Fig. 1c). A similar pattern of heterochromatin distribution on the autosomes was observed for *A. lituratus*, with large heterochromatic blocks on short arms of chromosome pairs 5^th^-7^th^ (Fig. 1d). However, the 1^st^ and 2^nd^ pairs showed only centromeric blocks. The X chromosome showed centromeric heterochromatin, as well as CH blocks on the long arms. Finally, the patterns of CH distribution on Y_1_ and Y_2_ chromosomes were similar to those of *A. planirostris*.

**Figure 2.**
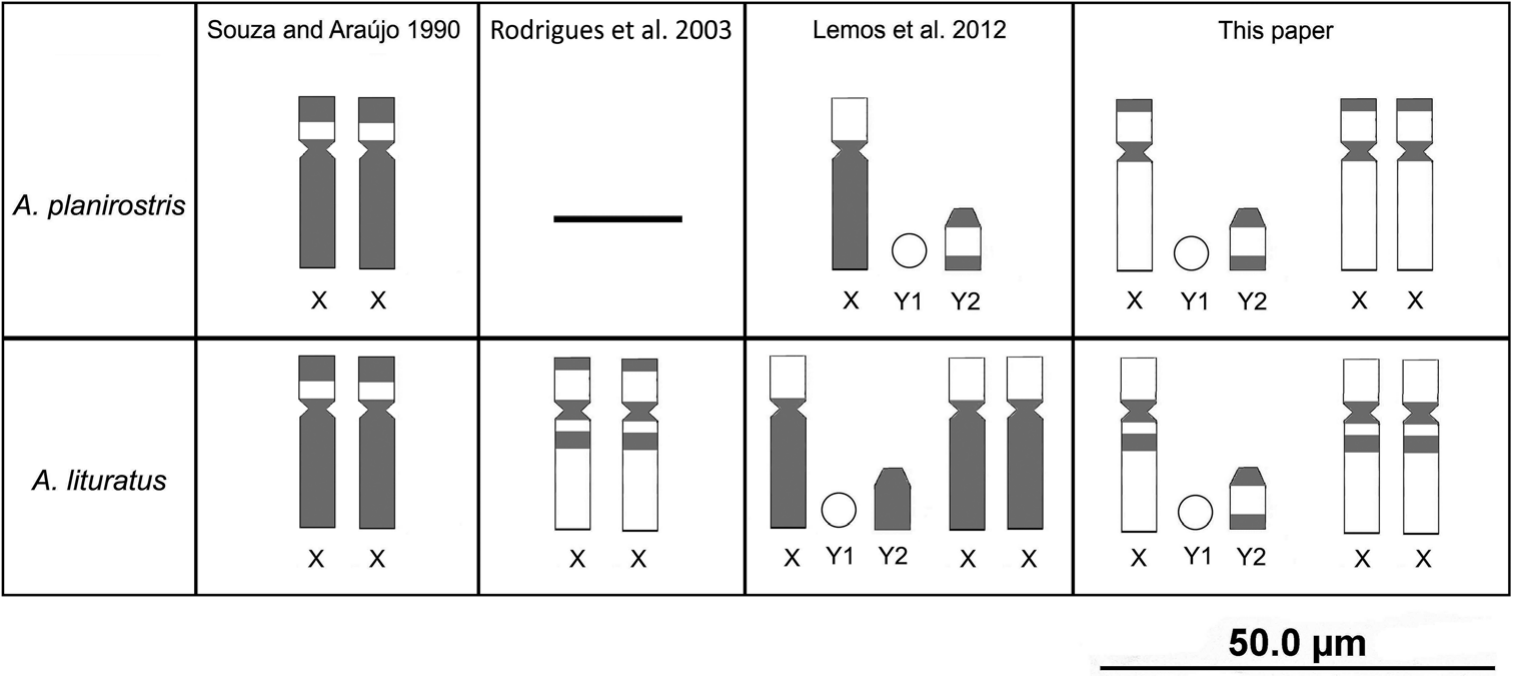
Schematic representation of *A. planirostris* and *A. lituratus* sex chromosomes showing C-banding variation reported in different studies. Gray shading corresponds to heterochromatin and the euchromatic regions are depicted in white color.

C-banding did not disclose within-species variation in our Amazonian samples. The observed CH patterns are, however, distinct from those reported in non-Amazonian indivuduals, indicating the existence of interindividual variation in both *Artibeus* species (Souza and Araujo 1990, Rodrigues et al. 2003, Lemos-Pinto et al. 2012). For example, we did not detect heterochromatic blocks on the distal region of the 9^th^ pair in individuals of either species as previously described by Lemos-Pinto et al. (2012) for samples collected in Pernambuco state. Furthermore, our results indicate that heterochromatin distribution on X chromosomes can vary not only among species, but also within species (Fig. 2). In this regard, two patterns were previously reported for specimens of *A. planirostris* from Pernambuco, Northeastern Brazil: (i) heterochromatic sites at centromeres, long arm, and distally on the short arm (Souza and Araújo 1990); and (ii) heterochromatic sites at the centromere and long arm (Lemos-Pinto et al. 2012). Both results differ from the data presented here because the long arm of X chromosomes of Amazonian specimens lacked evident CH blocks. Similarly, specimens of *A. lituratus* from Pernambuco have two patterns: (i) centromeric, plus distal on the short arm, and long arm (Souza and Araújo 1990); and (ii) centromere and long arm (Lemos-Pinto et al. 2012). Additionally, in specimens collected in Pará state (Northern Brazil), the X heterochromatin was centromeric, distal on the short arm, and interstitial on the long arm (Rodrigues et al. 2003).

Although the number of analyzed individuals (eight *A. planirostris* and three *A. lituratus*) from the Amazon is too low to make generalizations, the similar number and location of heterochromatic blocks between individuals from Pará and Amazonas (both Northern Brazil), might indicate that specimens from the same ecogeographic regions have similar CH patterns. Pará and Amazonas states are contiguous and covered mostly by Amazon rainforest, whereas Pernambuco is a coastal state, separated from the Amazon by dry forests, and transitional environments, which might serve as mild dispersion barriers. Therefore, additional studies including large sampling are required to test the hypothesis that CH variation occurs by differential turnover of repetitive DNA (derived either by their removal/amplification or by recombination), reinforced by geographical barriers through the distributional gradient of species.

### Ag-NOR Staining and In Situ Hybridization with Repetitive Probes

Silver nitrate staining and 18S rDNA FISH detected NORs at multiple sites on chromosomes of both species, more specifically distally on the short arms of the 5^th^, 6^th^ and 7^th^ pairs (Fig. 1e; data not shown for *A. lituratus*). The present Ag-NORs and 18S rDNA results agree with previously reported data (Morielle and Varella-Garcia 1988, Souza and Araújo 1990, Santos et al. 2002, Lemos-Pinto et al. 2012, Calixto et al. 2014). Many species in the subfamily Stenodermatinae have multiple NORs, which is considered a derivative condition. For example, this condition regards other *Artibeus* species (subtribe Artibeina H. Allen, 1898), *Uroderma bilobatum* W. Peters, 1866, *U. magnirostrum* Davis, 1968, *Vampyriscus bidens* (Dobson, 1878), and *Vampyressa thyone* O. Thomas, 1909 (subtribe Vampyressina Baker et al., 2016), as well as *Centurio senex* Gray, 1842 (subtribe Stenodermatina Gervais, 1856) (Baker et al. 1992, Santos et al. 2002, Gomes et al. 2016). However, the multiple NORs of the above mentioned groups are not necessarily located on homologous chromosomes (orthologous chromosome regions). Additionally, basal clades within Stenodermatinae (e.g., genus *Sturnira* Gray, 1842), and species in the same tribe as *Artibeus* (e.g., *Platyrrhinus* Saussure, 1860 and *Mesophylla* O. Thomas, 1901 species) do not have multiple NORs (Gomes et al. 2016). Therefore, we hypothesize that the presence of NORs on the three particular chromosome pairs of the analyzed species was a feature of the common ancestor of all *Artibeus* (Santos et al. 2002, Baker et al. 2016, Gomes et al. 2016). As another mammals, *Artibeus* NORs collocate (are adjacent) with heterochromatin and are likely associated with the amplification of heterochromatin in non centromeric regions.

In both species, *in situ* hybridizations detected (TTAGGG)_n_ telomeric sequences in all telomeres. Additionally, both species shared centromeric signals on three subtelocentric pairs (pairs 5^th^, 6^th^ and 7^th^; Fig. 1f, g). There are two potential explanations for the presence of telomeric sequences in interstitial position (ITS): (i) these sequences might be telomere motifs reallocated from the terminal region of a chromosome to another chromosome or chromosome position; and (ii) the ITS presence on the centromere derives from reorganization of repetitive sequences (satellite DNA) composing these regions, which could also indicate the presence of centromeric hotspots of recombination during *Artibeus* karyotype evolution (Nanda and Schmid 1994, Multani et al. 2001, Metcalfe et al. 2007, Faria et al. 2009, Kasahara 2009, Silva et al. 2016, Teixeira et al. 2016). The karyotypic evolution of *Artibeus* is considered extremely conservative, however the formation of the ancestral karyotype of the subfamily Stenodermatinae required extreme reshuffling (Baker and Bickham 1980, Pieczarka et al. 2013). Therefore, the ITS allocated on the pairs 5 ^th^–7 ^th^ for both species might be remnants of chromosome rearrangements that have been amplified or lost differentially in different Stenodermatinae species. Calixto et al. (2014) have shown that many phyllostomids species present ITS, regardless of their trend of karyotypic evolution. For example, species with a conservative karyotypic evolution, such as *Trachops cirrhosus* (Spix, 1823) and *Phyllostomus elongatus* (É. Geoffroy St. -Hilaire, 1810) (both in the subfamily Phyllostominae Gray, 1825) present ITS, which suggest lineage-specific events of amplification of these sequences can occur independently. Furthermore, it is noteworthy that pairs 5^th^–7^th^ have ITS, cetromeric and non-centromeric CH blocks, as well as the NORs in all *Artibeus* specimens analyzed, suggesting that differential dynamics of heterochromatin DNA in these particular chromosomes might have played a role in the establishment of their shared distinct architecture, when compared to other autosomes. Refined investigation of these chromosomes at the sequence level will help disclosing whether differential heterochromatin composition contributed to the establishment of centromeric ITS.

LINE-1 mapping on *A. planirostris* chromosomes revealed FISH signals near the centromere of most autosomes, except pairs 4, 7, 8, 13 and 14. (Fig. 1g). The centromeric FISH results were consistent in all analyzed individuals (n=4) and the centromeric pattern contrasts with the longitudinal distribution previously shown for most mammals, including other phyllostomid bats, *Carollia brevicauda* (Schinz, 1821), *Lophostoma occidentalis* (Davis & Carter, 1978), and *Gardnerycteris crenulatum* (É. Geoffroy St. -Hilaire, 1803) (Parish et al. 2002, Dobigny et al. 2006, Ferreri et al. 2011, Pieczarka et al. 2013, Sotero-Caio et al. 2015). Centromeric accumulation of retroelements in mammalian chromosomes is rare, but some cases have been described. For instance, Waters et al. (2004) found LINE-1-positive centromeres in the karyotypes of African mammals. Likewise, Sotero-Caio et al. (2015) showed centromeric LINE-1 accumulation in chromosomes of the phyllostomid bat *Tonatia saurophila* Koopman & Williams, 1951 (Phyllostominae). It was hypothesized that this unusual distribution might have contributed to the high degree of chromosomal reorganization in the genus *Tonatia* Gray, 1827. From our data, it is still premature to state that this retroelement or sequences derived from it are constitutional components of core centromeres. Similarly, because our probes comprised only a partial LINE-1 sequence, we cannot conclude that functional elements are contributing to the centromere dynamics of *Artibeus*. Despite the uncertainty on what factors were responsible for the massive “colonization” of LINE-1s at centromeres, processes such as gene conversion, which promote homogenization of centromeric sequences are expected to facilitate the maintenance of LINE-1 sequences in high copy numbers in this region (Shi et al. 2010).

We noticed inconsistent patterns when comparing the co-distribution of heterochromatin blocks and LINE-1 elements. Namely, in all analyzed individuals, interstitial CH blocks have LINE-1 signals in the second chromosome pair but not pair 1. Thus, non-centromeric heterochromatin formation on chromosomal arms of *A. planirostris* could be a result of amplification of different types of repeats (e.g. LINEs vs. satellite DNA) in specific chromosomes (Parish et al. 2002, Dobigny et al. 2006, Sumner 2008, Shi et al. 2010, Ferreri et al. 2011, Carbone et al. 2012).

The Y_1_ and Y_2_ sex chromosomes presented weak FISH signals, contrasting with the strong signal throughout the long arm of the X (Fig. 1h). LINE-1 accumulation on X chromosomes is a pattern observed in all mammal species, including other phyllostomid bats (Lyon 1998, Parish et al. 2002, Dobigny et al. 2006, Cantrell et al. 2008, Liu et al. 2011, Sotero-Caio et al. 2015). Parish et al. (2002) investigated the concentration of LINE-1 in *C. brevicauda* chromosomes, which also presents a multiple sex chromosome system. They found that the original X chromosome had higher levels of LINE-1 accumulation than the translocated autosome. The X-autosome translocation of *Artibeus* is different from that observed in *C. brevicauda*, with the small autosome component representing the short arm of *A. planirostris* X chromosome. In agreement with Parish et al. (2002) findings and Lyon hypothesis, we identified that Xq of *A. planirostris* had a significant accumulation of LINE-1, corresponding exactly to the original X chromosome. The LINE-1 accumulation on Y-chromosomes seems to be restricted to centromeric regions. In this case, we expect that similarly to other mammals, the original Y is mostly constituted of repeats other than retroelements, and that Y_2_ pattern corresponds to that observed for other autosomes due to its autosomal origin.

Transposable element activity and accumulation have been linked to chromosomal rearrangements and can be directly or indirectly associated with speciation events (Lim and Simmons 1994, Dörner and Pääbo 1995, Gray 2000, Dobigny et al. 2004, Waters et al. 2004, Carbone et al. 2012, 2014). In addition, the dispersal dynamics of TEs are related to biological functions such as gene regulation, chromosomal rearrangements, X inactivation on females and horizontal transfer events among closely or distantly related species (Lyon 1998, Ostertag and Kazazian Jr 2001, Chow et al. 2010) . The distribution of repetitive elements in *Artibeus* might have played a significant role in shaping the chromosome architecture of the genus, and we are still unsure if this trend at centromeres can be observed in other species of Stenodermatinae. Because the LINE-1 accumulation patterns differ in the bat species analyzed to date (Parish et al. 2002, Sotero-Caio et al. 2015, present study), we hypothesize that these elements constitute potential contributors to the great karyotype reshuffling presented by some phyllostomid taxa since the divergence of their ancestral karyotype. Overall, our data suggest that different mechanisms might have contributed to the karyotype evolution of phyllostomid bats, explaining why only *Artibeus* and *Tonatia* species, but not *C. brevicauda*, *L. occidentalis*, and *G. crenulatum* differred in the patterns of LINE-1 distribution.
